# Centaurin-α_2_ Interacts with β-Tubulin and Stabilizes Microtubules

**DOI:** 10.1371/journal.pone.0052867

**Published:** 2012-12-20

**Authors:** Paola Zuccotti, Daniele Cartelli, Michela Stroppi, Vittorio Pandini, Marco Venturin, Alessandro Aliverti, Elena Battaglioli, Graziella Cappelletti, Paola Riva

**Affiliations:** 1 Dipartimento di Biotecnologie Mediche e Medicina Traslazionale, Università degli Studi di Milano, Milano, Italy; 2 Dipartimento di Bioscienze, Università degli Studi di Milano, Milano, Italy; Stanford University School of Medicine, United States of America

## Abstract

Centaurin-α_2_ is a GTPase-activating protein for ARF (ARFGAP) showing a diffuse cytoplasmic localization capable to translocate to membrane, where it binds phosphatidylinositols. Taking into account that Centaurin-α_2_ can localize in cytoplasm and that its cytoplasmatic function is not well defined, we searched for further interactors by yeast two-hybrid assay to investigate its biological function. We identified a further Centaurin-α_2_ interacting protein, β-Tubulin, by yeast two-hybrid assay. The interaction, involving the C-terminal region of β-Tubulin, has been confirmed by coimmunoprecipitation experiments. After Centaurin-α_2_ overexpression in HeLa cells and extraction of soluble (αβ dimers) and insoluble (microtubules) fractions of Tubulin, we observed that Centaurin-α_2_ mainly interacts with the polymerized Tubulin fraction, besides colocalizing with microtubules (MTs) in cytoplasm accordingly. Even following the depolimerizing Tubulin treatments Centaurin-α_2_ remains mainly associated to nocodazole- and cold-resistant MTs. We found an increase of MT stability in transfected HeLa cells, evaluating as marker of stability the level of MT acetylation. In vitro assays using purified Centaurin-α_2_ and tubulin confirmed that Centaurin-α_2_ promotes tubulin assembly and increases microtubule stability. The biological effect of Centaurin-α_2_ overexpression, assessed through the detection of an increased number of mitotic HeLa cells with bipolar spindles and with the correct number of centrosomes in both dividing and not dividing cells, is consistent with the Centaurin-α_2_ role on MT stabilization. Centaurin-α_2_ interacts with β-Tubulin and it mainly associates to MTs, resistant to destabilizing agents, in vitro and in cell. We propose Centaurin-α_2_ as a new microtubule-associated protein (MAP) increasing MT stability.

## Introduction

Human Centaurin-α_2_, recently renamed “ARFGAP protein with dual PH (pleckstrin homology) domain-containing protein 2” (*ADAP2*) to stress the systematic relationships within the superfamily of ARFGAP proteins, is also characterized by a C4-type zinc finger and two PH domains. Centaurin-α_2_, together with −α_1_, −β, −γ and −δ, constitutes the Centaurin family characterized by the presence of at least a PH domain, ankyrin repeats and a conserved Cx_2_Cx_16–17_Cx_2_C domain, which acts as a zinc-binding motif [1]. Centaurin-α_2_ has been shown to bind phosphatidylinositol-trisphosphate *in vitro* [2]. It has a diffuse cytoplasmic localization and has been found to translocate, after Epidermal growth factor (EGF) stimulation, from the cytoplasm to the plasma membrane, where it preferentially binds phosphatidylinositol-3,4-bisphosphate (PIP_2_), through its C-PH domain [3]. Centaurin-α_2_ displays a GTPase-activating protein activity on the ADP-ribosylation factor 6 (ARF6), a small GTPase involved in actin cytoskeleton remodelling. Plasma membrane association of Centaurin-α_2_ prevents ARF6 translocation to, and cortical actin formation at, the plasma membrane and thereby negatively regulates ARF6-mediated cytoskeleton actin reorganisation [3]. However the distribution of Centaurin-α_2_ in intact cells is predominantly cytosolic and, in this context, its biological function is not known.

Centaurin-α_2_ expression pattern reveals high expression levels in placenta, spleen, kidney, skeletal muscle and adrenal gland and weak signals in thyroid, liver, heart, lung, small intestine, peripheral blood leucocytes [2]. During human foetal development *ADAP2* was found to be expressed in skeletal muscle, liver and brain with a high expression in heart and aorta, and it has been detected in heart and brain during the first phases of mouse embryonic development [4]. It has been recently observed, by *in situ* hybridizations on mouse embryo, that *Adap2* is expressed in early stages of heart development (9 dpc), during the formation of cardiac camera, septa and valves (M. Venturin, personal communication). Interestingly *ADAP2* gene was one of the genes found to be deleted in NF1 microdeletion patients showing a high incidence of cardiovascular malformations, most of which are valve or atrial/ventricular septa defects [5]. This evidence strongly suggests that *ADAP2* can be a candidate gene for these specific heart abnormalities.

In an attempt to clarify the biological functions of this protein, we searched for cytosolic Centaurin-α_2_ interactors. Here we report on Centaurin-α_2_-β-Tubulin interaction, where Centaurin-α_2_ was found to be mainly associated to the β-Tubulin polymerized form increasing MT stability. Functional studies indicated that Centaurin-α_2_ stabilizes MTs with a role in the correct mitotic spindle formation. The obtained findings are strongly indicative that Centaurin-α_2_ is a new MAP.

## Results

### Centaurin-α_2_ interacts with β-Tubulin

**Figure 1:**
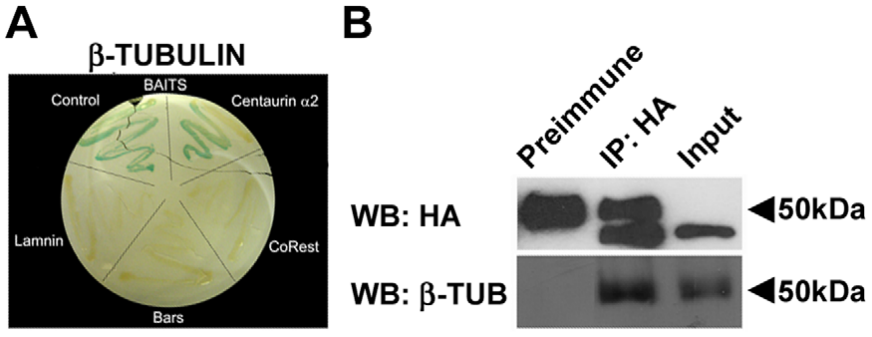
Centaurin-α_2_ interacts with β-Tubulin. **A**) Yeast two-hybrid assay on L40 yeast cotransformed with Tubulin β chain and different baits (pSTT91-Centaurin-α_2_, pBTM116-CoRest, pBTM116-laminin or pBTM116-bars. **B**) Immunoprecipitation of β-Tubulin Centaurin-α_2_. Immunoblot of Centaurin-α_2_ (HA, upper panel) and of β-Tubulin (β-Tub, lower panel) were performed on total extracts from HeLa cells transfected with pCGN-Centaurin-α_2_ (Input), and on extracts immunoprecipitated with anti-HA antibody (IP:HA) and Preimmune serum.

With the aim of identifying novel proteins interacting with human Centaurin-α_2_, a yeast two-hybrid assay has been carried out using as bait a fusion protein between LexA DNA binding domain and the full length human Centaurin-α_2_. Following the exclusion of the auto-activation of β-galactosidase gene by the bait, avoiding false positives (see Materials and Methods), L40 yeast has been cotransformed with pSST91-Centaurin-α_2_ and a human foetal brain cDNA library. A screening of 1.2×10^6^ cotransformants has led to the isolation of 36 positive clones growing on the selective medium -Leu –Trp –His positive at the β-galactosidase assay, in which two known interacting proteins CoRest-Kiaa0601 [6] have been used as positive control. The 36 positive clones have been shown to be unable to activate transcriptional machinery by means of β-galactosidase assay after extraction of the bait from transformed yeast. Following sequencing of each clone, six possible interactors have been identified. For all of them the interaction specificity for the Centaurin-α_2_-bait has been tested by using as baits unrelated control proteins, such as CoRest, Bars and Laminin. Only the construct encoding for Tubulin β chain class I (NP_821133.1), showed a specific interaction with Centaurin-α_2_-bait (Figure 1 A).

The Tubulin, β cDNA has been found to encode for the last 116 aminoacids of the C-terminal protein region and to include part of the 3′UTR. Tubulin β chain class I isotype is ubiquitously expressed, sharing at least 95% of the aminoacid sequence with the other isotypes, with the exception of the class VI isotype sharing the 80% of the sequence. Tubulin β chain, from now defined by the term β-Tubulin, is present in cells both in soluble dimeric form, associated with α-Tubulin, and in polymerized form constituting MTs.

To confirm the interaction between Centaurin-α_2_ and Tubulin β, coimmunoprecipitation (CoIP) experiments have been carried out. HeLa cells, expressing low endogenous levels of Centaurin-α_2_, (Figure S1) were transfected with pCGN-Centaurin-α_2_, a vector encoding for HA (hemoagglutinin)-tagged Centaurin-α_2_. Total cell lysates were immunoprecipitated with anti-HA antibody or a pre immune IgG serum (PI) and analyzed by western blotting. The anti-HA antibody identified a band with the apparent molecular weigth of 44 kDa corresponding to the recombinant HA-Centaurin-α_2_, both in the immunoprecipitation and in the input lanes (Figure 1 B). Note that, due to cross-reaction of the HA antibody with the IgG, both in the IP and in the pre immune (PI) lane is present at about 50 kDa, a non specific band. After incubation with monoclonal anti-β-Tubulin antibody, a band of about 50 kDa has been detected in IP lane as well as in the input lane (Figure 1 B). The presence of a band corresponding to β-Tubulin in the HA-IP lane shows that Tubulin coimmunoprecipitates with Centaurin-α_2_, indicating that the interaction among Centaurin-α_2_ and β-Tubulin can occur *in vivo* in mammalian cells.

### Centaurin-α_2_ interacts with Tubulin preferentially associating with the polymerized Tubulin fraction in mammalian cells

**Figure 2:**
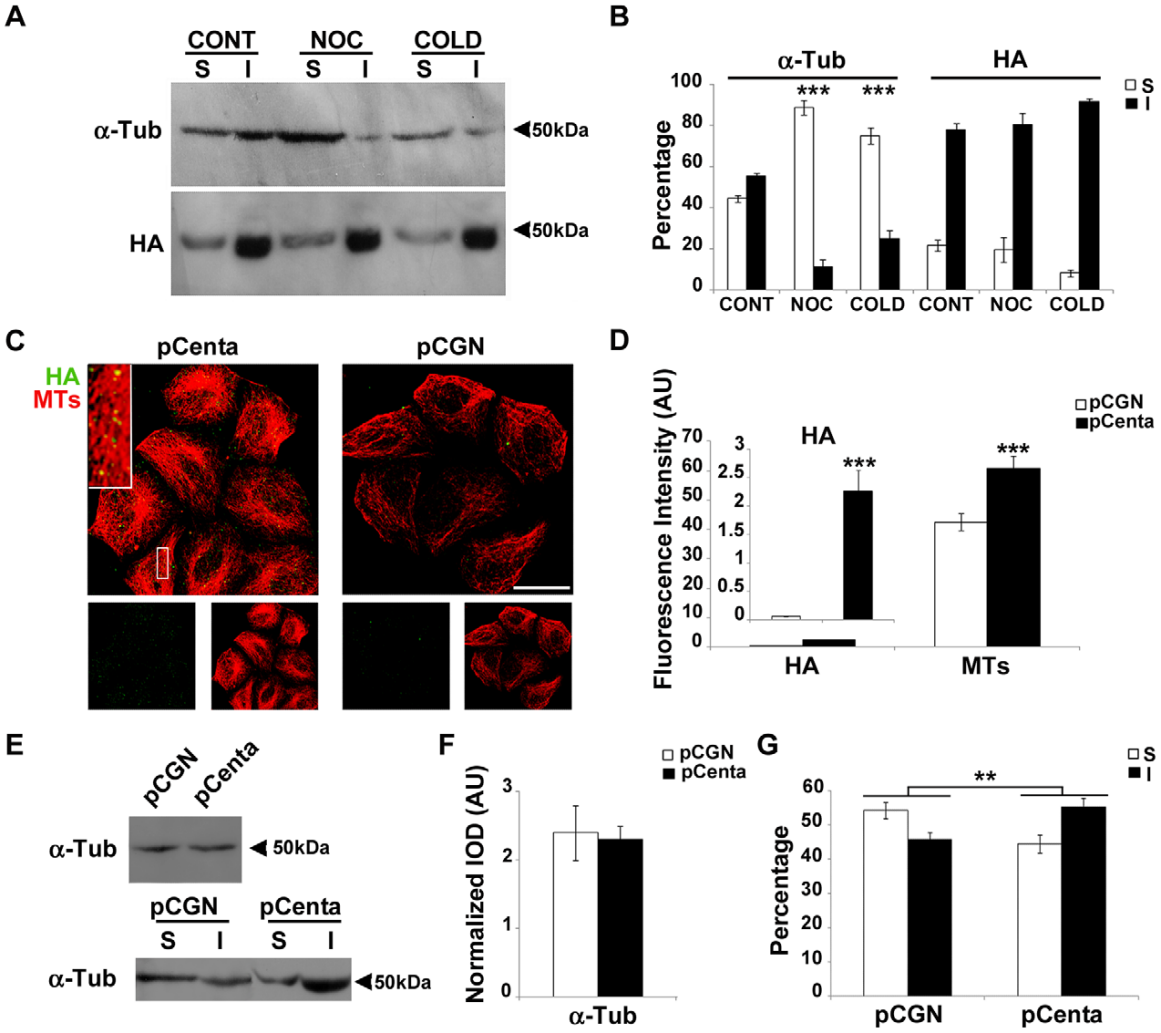
Centaurin-α_2_ binds to stable MTs in HeLa cells. Immunoblots (**A**) and densitometric analyses (**B**) of Triton-soluble (S, white bars) and insoluble (I, black bars) fractions of pCGN-Centaurin-α_2_ transfected HeLa cells, in control conditions (CONT) or treated with nocodazole (NOC) and cold (COLD). ***p<0.005 vs CONT. **C**) Cytoskeletal fractions of Hela cells transfected with pCGN-Centaurin-α_2_ or with pCGN vector were immonustained with anti-HA (green) and anti-α-Tubulin (red) antibodies. For pCGN-Centaurin-α_2_ merged image, the inset (white rectangle) show the discrete distribution of HA spots along microtubules. Scale bar: 20 µm. **D**) Analyses of the intensity of fluorescence of green signal (HA) and red signal (MTs) in pCGN (white bars) or pCGN-Centaurin-α_2_ (black bars) expressing HeLa cells. ***p<0.005 vs pCGN. **E**) Immunoblots of the total and free (S) or polymerized (I) α-Tubulin (α-Tub) and densitometric analyses of whole cell extracts (**F**) or Triton-soluble (S, white bars) and insoluble (I, black bars) fractions (**G**) of pCGN (pCGN) or pCGN-Centaurin-α_2_ (pCenta) transfected HeLa cells. **p<0.01 vs pCGN.

To establish if Centaurin-α_2_ interacts with Tubulin dimers or with MTs in cells, we analyzed cytoskeletal fractions obtained from pCGN-Centaurin-α_2_ transfected HeLa cells following Triton-X-100 extraction. By western blotting and densitometric analysis, we evaluated the percentage of both Tubulin and Centaurin-α_2_ associated with Triton-soluble (S) and -insoluble (I) fraction. As shown in Figure 2 A and B, Tubulin is similarly distributed between soluble (dimers) and insoluble (MTs) fractions as expected. Conversely, the greater part of Centaurin-α_2_ (80%) is found in the insoluble fraction, suggesting that Centaurin-α_2_ preferentially interacts with MTs, whereas the remaining 20% found in the soluble fraction could be associated with Tubulin dimers or could be free.

We therefore investigated if Centaurin-α_2_ binds to stable or labile MTs, resistant to destabilizing treatments or prone to depolymerization, respectively. To pursue this scope we challenged HeLa cells with well known MT-destabilizing treatments, such as nocodazole and cold. As expected, MTs significantly depolymerise following both treatments, and the vast majority of Tubulin is recovered in the dimeric fraction (Figure 2 A and B). On the contrary, most of Centaurin-α_2_ remains associated with the Triton-insoluble fraction, highlighting that Centaurin-α_2_ binds to nocodazole- and cold-resistant MTs.

To confirm the Centaurin-α_2_ binding to Tubulin insoluble fraction, we transfected HeLa cells with pCGN-Centaurin-α_2_ or with the pCGN vector encoding only the HA-tag and removed the Triton-soluble fraction. Then, we double stained the Triton-insoluble fraction with anti-α-Tubulin and anti-HA antibodies, and we analyzed the samples by confocal microscopy. As shown in Figure 2 C, there is no evidence of Centaurin-α_2_ aggregation; instead it appears as discrete punctae decorating MTs, meaning that Centaurin-α_2_ behaves like a MT interacting protein, remaining attached to MTs also after the removal of unassembled Tubulin. HeLa cells transfected with the control vector show very few HA spots, and the analyses of fluorescence intensity (Figure 2 D) report a significant lower HA fluorescence in pCGN transfected cells than in Centaurin-α_2_ expressing HeLa, allowing us to exclude a tag-mediated binding and supporting the in cell interaction between Centaurin-α_2_ and MTs. These results are reinforced by the analyses of colocalization parameters (reviewed in [7]).

The Pearson's coefficient (Table 1) together with the fluorograms (Figure S2) are rough estimations of the association between the fluorescence signals, and therefore they could show the association between the two proteins; indeed, our analyses reveal that the presence of Centaurin-α_2_ significantly increases the colocalization between HA and Tubulin signals reinforcing the idea of an interaction between the two proteins. Furthermore, the evaluation of Manders' coefficients (Table 1), representing the fraction of a signal superimposed to the other, highlights a significantly higher overlapping of both HA-Centa over Tubulin (M1) and Tubulin over HA-Centa (M2) compared to the empty vector (pCGN). Altogether, these analyses strengthen the idea of a direct binding of Centaurin-α_2_ to Tubulin in cell.

**Table 1:** Analyses of colocalization between Centaurin-α_2_ and MTs.

	pCGN	pCenta	*p* Value
**Pearson**	0.017±0.003	0.164±0.063	<0.05
**M1** (HA vs Tub)	0.61±0.03	0.71±0.02	<0.03
**M2** (Tub vs HA)	0.01±0.003	0.21±0.05	<0.001

The *p* values are calculated according to the Student's t-test.

Interestingly, we observed that, Centaurin-α_2_ overexpressing cells show a significant increase in Tubulin fluorescence in respect to control cells. To verify if the higher Tubulin signal was due to an increased MT mass, we performed western blot analysis after extraction of total protein and of soluble/insoluble fractions in both pCGN-Centaurin-α_2_ and pCGN transfected HeLa cells. The obtained results indicate that Centaurin-α_2_ overexpression does not induce any increase of total Tubulin level (Figure 2 E and F), while the distribution of Tubulin significantly shifts from the soluble to the insoluble pool in Centaurin-α_2_ transfected cells compared to the control (Figure 2 E and G). Taken together, these results suggest that Centaurin-α_2_ binding to MTs promotes the increase of the MT fraction and concomitant depletion of the free Tubulin pool, suggesting that Centaurin-α_2_ enhances MT stability.

### Pure Centaurin-α_2_ increases microtubule formation and stability in vitro

**Figure 3:**
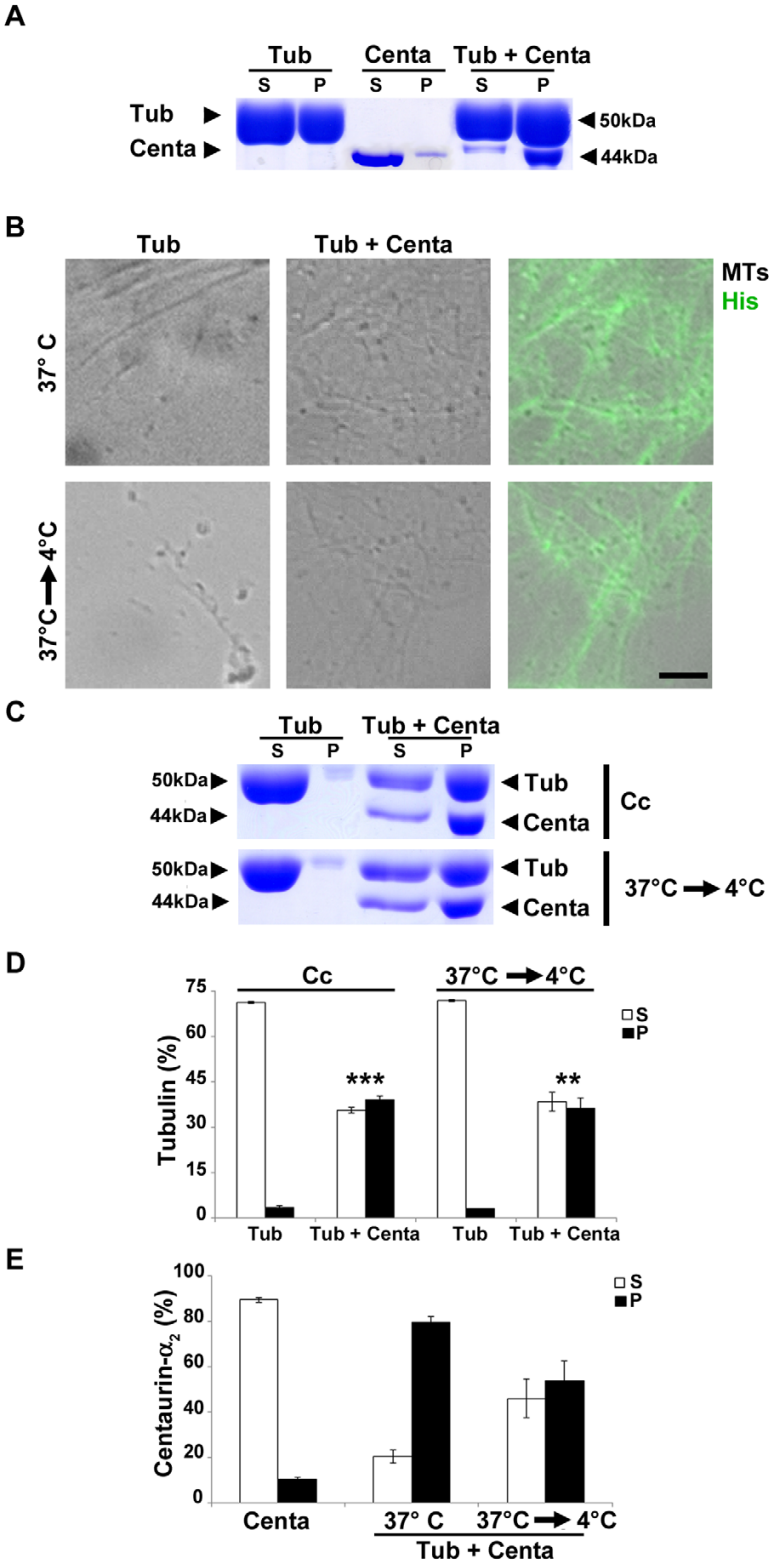
Centaurin-α_2_ increases MT formation and stability. A) Comassie Blue stained SDS-PAGE gel of supernatant (S) and pellet (P) fractions of 40 µM Tubulin (Tub), 5 µM Centaurin-α_2_ (Centa) or 40 µM Tubulin plus 5 µM Centaurin-α_2_ (Tub+Centa) after 90 minutes of polymerization at 37°C. **B**) Anti-His staining (Green) on *in vitro* MTs assembled in the absence (Tub) or in the presence of 5 µM Centaurin-α_2_ (Tub + Centa), w/o (37°C) or with (37°C→4°C) 30 minutes of incubation on ice. MTs were visualized by DIC microscopy. Scale bar: 2 µm. Comassie Blue stained SDS-PAGE gel (**C**) and densitometric analyses of tubulin content (**D**) of supernatant (S, white bars) and pellet (P, black bars) fractions of MTs polymerized at 18 µM tubulin (Cc) or at 40 µM and then destabilized 30 minutes on ice (37°C→4°C), in the absence (Tub) or in the presence of 5 µM Centaurin-α_2_ (Tub + Centa). **p<0.02, ***p<0.005 vs Tub. **E**) densitometric analyses of Centaurin-α_2_ associate to supernatant (S, white bars) and pellet (P, black bars) fractions of 5 µM Centaurin-α_2_ (Centa) or 40 µM Tubulin plus 5 µM Centaurin-α_2_ (Tub+Centa) after 90 minutes of polymerization at 37°C (37°C) or after 30 minutes of incubation on ice (37°C→4°C).

To better characterize the interaction between Centaurin-α_2_ and MTs, we moved to experiments with pure proteins, performing polymerization of bovine brain tubulin in the presence of His-tagged Centaurin-α_2_ (Figure 3). At the end of the assembly, tubulin dimers (S) and MTs (P) were separated by centrifugation and resolved on SDS-PAGE and, as shown in Figure 3A, Centaurin-α_2_ alone is almost completely recovered in the supernatant whereas, in the presence of Tubulin, 80% of Centaurin-α_2_ is associated to MT fraction (Figure 3 E). Therefore, this approach allowed us to demonstrate that Centaurin-α_2_ mainly interacts with MTs. To verify whether true MTs are forming in the presence of Centaurin-α_2_, we used DIC microscopy. Figure 3 C (top) shows that Centaurin-α_2_ promotes the formation of MTs and, as revealed by anti-His immunofluorescence, it is associated to MTs. Following MT-destabilization by cold treatment (30 minutes of incubation on ice), we investigated if Centaurin-α_2_ binds to stable or labile MTs. This approach strongly suggests that Centaurin-α_2_ protects MTs from cold treatment and that it remains associated to cold-resistant MTs (Figure 3 C, bottom); indeed, after 30 minutes on ice, around 50% of Centaurin-α_2_ is still recovered in the MT fraction (Figure 3 C and E). Finally, to verify that pure Centaurin-α_2_ is able to promote MT formation and stabilization we used two different approaches (Figure 3 C and D). We performed MT assembly assay using an amount of tubulin around the critical concentration (Cc), i.e. the minimal concentration allowing MT formation (17.85 µM for our tubulin batch). In control conditions a low level of tubulin was present in the polymerized fraction (P), whereas Centaurin-α_2_ strongly increased MT assembly (Figure 3 C and D). Moreover, using standard concentration of tubulin and allowing MT disassembly (30 minutes of incubation on ice at the end of polymerization), we recovered a significant higher amount of MTs in the presence of Centaurin-α_2_, meaning that Centaurin-α_2_ protects MTs against cold-induced depolimerization. Altogether, these data show that Centaurin-α_2_ directly interacts with MTs, promoting MT formation and stabilization, *in vitro* and in cell as well.

### Centaurin-α_2_ increases microtubule acetylation

Nocodazole- and cold-resistant MTs are the most stable ones, and MT stability is regulated by Tubulin posttranslational modifications. A typical marker of dynamic MTs is the tyrosinated form of α-Tubulin that represents also the newly synthesized Tubulin, whereas more stable MTs are typically associated with the acetylation of α-Tubulin [8]. Interestingly, a protein binding nocodazole resistant MTs, named Dual specificity protein phosphatase CDC14B, is reported to enhance MT stability through the increase of Tubulin acetylation [9]. Similarly we investigated whether Centaurin-α_2_ mediated changes in MT stability through modulation of Tubulin posttranslational modifications.

**Figure 4:**
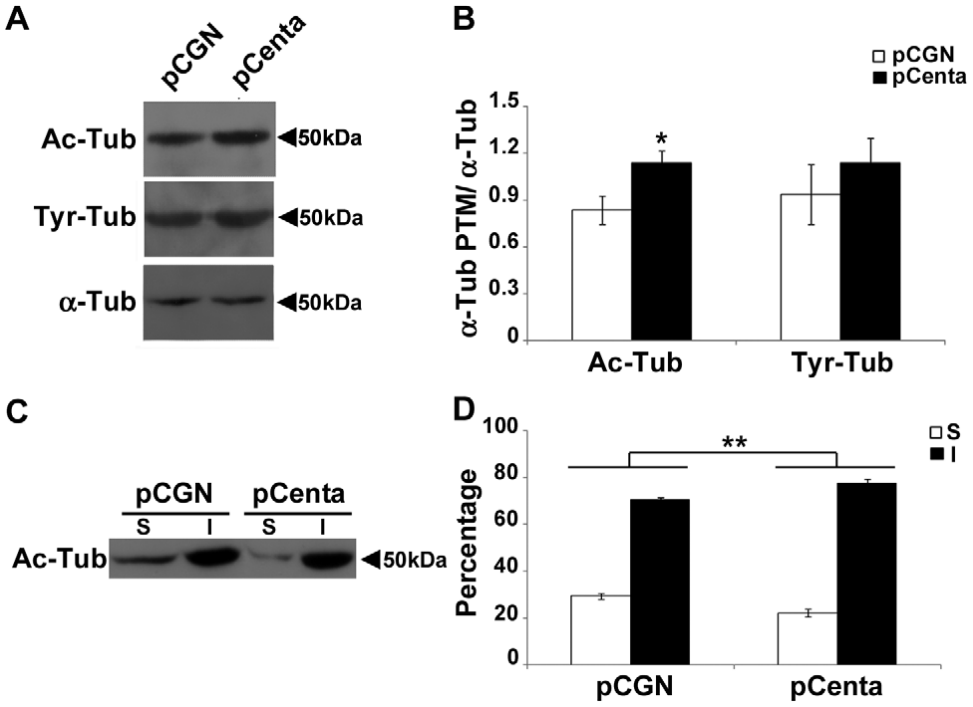
Centaurin-α_2_ increases MT acetylation. Immunoblot (**A**) and densitometric analyses (**B**) of acetylated-α-Tubulin (Ac-Tub) and tyrosinated-α-Tubulin (Tyr-Tub) in whole-cells extracts derived from HeLa cells transfected with pCGN (pCGN, white bars) or pCGN-Centaurin-α_2_ (pCenta, black bars). *p<0.05 vs pCGN. Immunoblots (**C**) and densitometric analyses (**D**) of acetylated-α-Tubulin (Ac-Tub) associated to Triton-soluble (S, white bars) and insoluble (I, black bars) of pCGN (pCGN) or pCGN-Centaurin-α_2_ (pCenta) expressing HeLa cells. **p<0.02 vs pCGN.

A western blotting and densitometric analysis on total extracts of HeLa cells transfected with either pCGN-Centaurin-α_2_ or pCGN, have been carried out by using antibodies specific for acetylated Tubulin, tyrosinated Tubulin and total α-Tubulin. We found that both tyrosinated and acetylated Tubulin increases in Centaurin-α_2_ overexpressing cells (Figure 4 A and B), even if only the Tubulin acetylation level is significantly higher, while total Tubulin level is the same in both Centaurin-α_2_ overexpressing and control cells. This finding together with the observation of a higher level of acetylated MT fraction in pCGN-Centaurin-α_2_ transfected cells compared to the control (Figure 4 C and D), is consistent with the above reported role of Centaurin-α_2_ in stabilizing MTs.

### Centaurin-α_2_ promotes the proper assembly of mitotic apparatus

**Figure 5:**
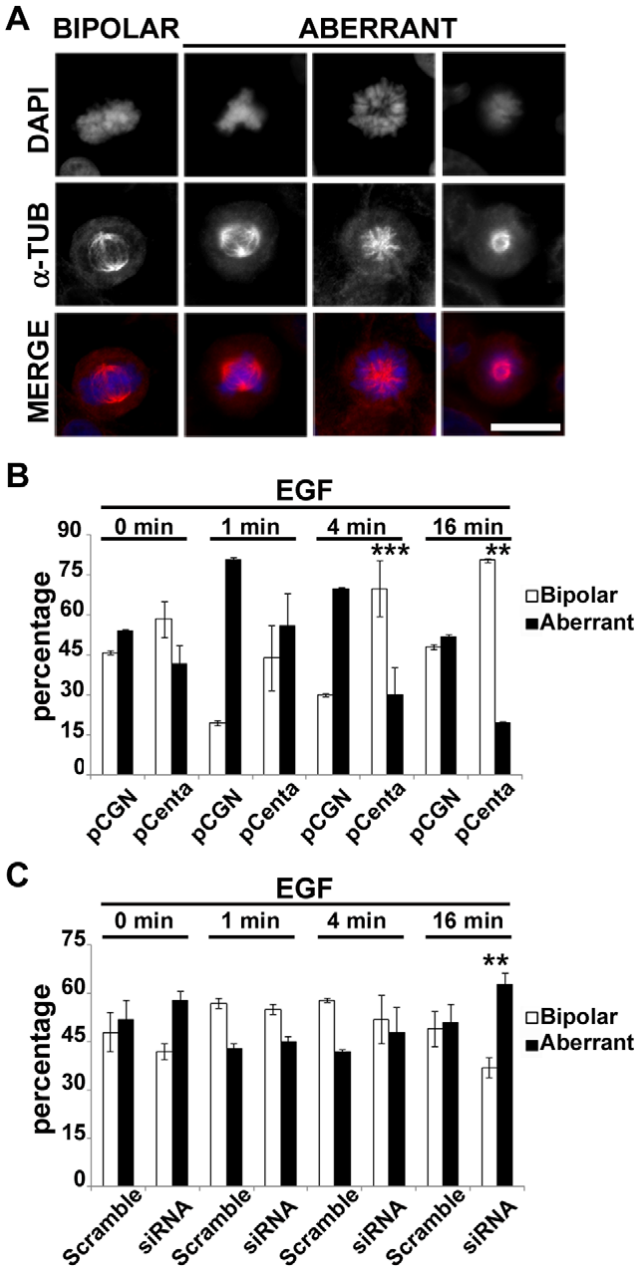
Centaurin-α_2_ promotes the correct assembly of mitotic apparatus. **A**) Representative microphotographs of the spindle morphologies observed in pCGN or pCGN-Centaurin-α_2_ expressing HeLa cells. **B**) Quantification of the percentage of cells displaying bipolar (white bars) or aberrant (black bars) spindles, after pCGN (pCGN) or pCGN-Centaurin-α_2_ (pCenta) transfection. **C**) Quantification of the percentage of cells displaying bipolar (white bars) or aberrant (black bars) spindles, after ON-TARGET plus SMART pool siRNA Centa2 or ON-TARGET plus Non-targeting Pool transfection, *p<0.05, **p<0.02, ***p<0.005 vs pCGN. Scale bar = 20 µm.

**Figure 6:**
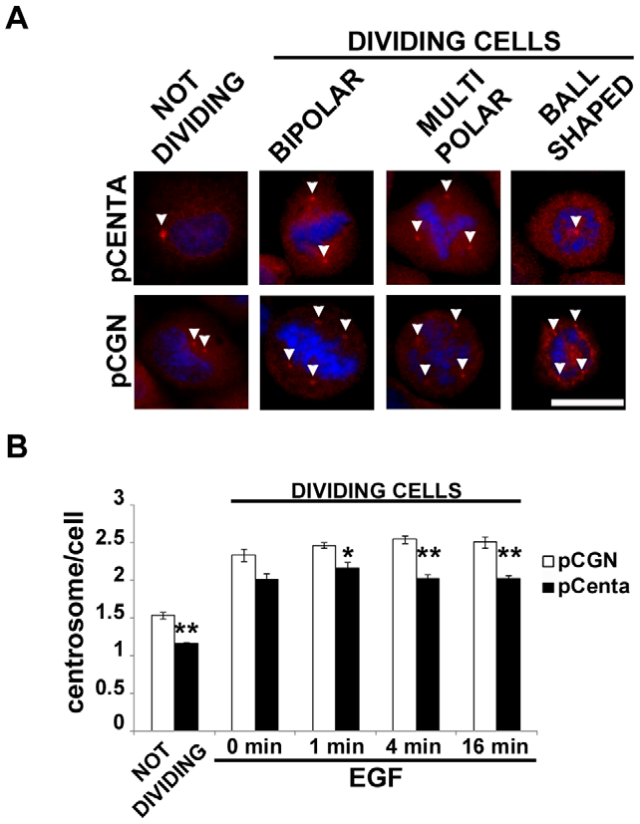
Centaurin-α_2_ decreases the HeLa cells with abnormal centrosome number. Representative microphotographs (**A**) and quantification (**B**) of centrosomes numbers associate to the pCGN (pCGN, white bars) or pCGN-Centaurin-α_2_ (pCenta, black bars) expressing HeLa cells, in not dividing and dividing cells. *p<0.05, **p<0.02 vs pCGN. Scale bar = 20 µm.

The control of MT stabilization is crucial for the functionality of MT machinery and the correct expression of its biological functions. It is reported that more stable MTs, i.e. more acetylated MTs, confer resistance to breakdown of the mitotic apparatus [10]. Given that Centaurin-α_2_ overexpression induced changes in MT stability, we wondered whether Centaurin-α_2_ was implicated in mitotic apparatus assembly. pCGN-Centaurin-α_2_ and pCGN transfected HeLa cells have been serum starved for 24 hours and stimulated with EGF for 0, 1, 4, 16 minutes to induce proliferation. After decoration of MTs with anti-α-Tubulin antibodies and chromosomes with DAPI, transfected and control cells show normal bipolar spindles but also aberrant forms, such as multipolar, characterized by plus than two centrosomes, and monopolar, with MTs radiating toward chromatin organized in a rosette pattern and a central polar area containing centrioles [11] (Figure 5 A). Both in stimulated and non stimulated cells an increasing of bipolar spindles in Centaurin-α_2_ overexpressing cells compared to control was detected with a statistically significant difference after 4 and 16 minutes of EGF stimulation (Figure 5 B). To verify whether the Centaurin-α_2_ downregulation lead to a further increase of abnormal mitotic spindle, HeLa cells were transfected with *ADAP2* specific-siRNA. The *ADAP2* mRNA level decreased up to 41% at 48 hours after cell transfection with 50 nM *ADAP2* siRNA. According to the treatments previously applied on Centaurin-α_2_ overexpressing cells, the number of abnormal spindle significantly increased after 16 minutes of EGF stimulation in respect to the control cells transfected with scrambled siRNA sequences (Figure 5 C). To elucidate the role of Centaurin-α_2_ in controlling the mitotic apparatus assembly we also evaluated the centrosome number in HeLa cells overexpressing Centaurin-α_2_ stimulated with EGF, both in interphase and mitosis. After a costaining with γ-Tubulin antibody and DAPI (Figure 6 A) we found that the number of centrosomes significantly decreases in Centaurin-α_2_ overexpressing cells compared to the control, both in interphase and in metaphase after EGF stimuli (Figure 6 B).

Together these data show that Centaurin-α_2_ is able to regulate the correct assembly of mitotic apparatus, probably with a role in centrosomes duplication and modulation of MT stability.

## Discussion

Centaurin-α_2_ is an ARFGAP protein showing a diffuse cytoplasmic localization capable to translocate to membrane, where it binds phosphatidylinositols, and displaying a GTPase-activating protein activity towards ARF6 [3]. Taking into account that Centaurin-α_2_ can localize in cytoplasm and that its cytoplasmatic function is not well defined, we searched for further interactors by yeast two-hybrid assay to investigate its biological function. Starting from a human foetal brain cDNA library, we identified the interaction between Centaurin-α_2_ and β Tubulin by yeast two hybrid assay and confirmed it by coimmunoprecipitation, in vitro assays using purified proteins and confocal microscopy in cells overexpressing Centaurin-α_2_. Furthermore, we showed that Centaurin-α_2_ is mostly associated to MTs, and maintained such an interaction also after depolymerizing treatments, i.e. cold treatment, suggesting a strong binding to MTs.

The Tubulin β interactor identified in this study is a β-Tubulin isotype expressed ubiquitously and at high levels in cells. As the prey clone contains the C-terminal portion of Tubulin β chain, corresponding to the hypervariable part of the protein characterizing specific isotypes [12], [13], [14], we were able to identify the β-Tubulin isotype and in particular the region interacting with Centaurin-α_2_. The isolated prey clone encodes the last 116 aminoacids of the C-terminal region, including two α-helices, H11 and H12, that are known to constitute the external surface of MTs involved in the binding with motor proteins and MAPs (microtubule associated proteins) and seems to interact with the α-Tubulin subunit [13], [14]. Further possible Centaurin-α_2_ interactors have been reported: ARF6 [3], Elongation factor 1-gamma [15], [16], RanBPM (Ran binding protein in microtubule-organizing center) [17], Nucleolin [17]. All these proteins with the exception of the Elongation factor 1-gamma have been proposed as “candidate interactors” as they were demonstrated to bind Centaurin-α_1_ [16], [17], [18], showing a high sequence homology with Centaurin-α_2_ [19]. As far as the Elongation factor 1-gamma, it has been identified by an automated yeast two-hybrid assay, where the pool of preys did not include the Tubulin β chain isotype. On the other hand, the experimental approach used in our study did not allow to fish the above described Centaurin-α_2_ interactors probably due to their enzymatic nature and their consequent transient binding to Centaurin-α_2_, as in the case of ARF6 and Nardilysin [3], [16], or due to a restricted spatio-temporal binding, as in the case of RanBPM [17].

We found that Centaurin-α_2_ mostly binds to the polymerized Tubulin, resistant to destabilizing agents, indicating a strong association to stable MTs. This finding has been supported by data concerning the *in vitro* experiments and the co-localization of Centaurin-α_2_ and MTs after immunostaining and confocal microscopy analysis, indicating that it behaves like a MT interacting protein. Furthermore, the experiments with pure proteins revealed that Centaurin-α_2_ acts with a taxol-like mechanism, promoting MT assembly in constrained conditions, i.e. near tubulin critical concentrations, and protecting MTs from cold-induced destabilization. These data agree with the results we obtained in cell, indeed an increased amount of polymerized Tubulin has been detected both *in vitro* and at cellular level. Together, the interaction with the C-term portion of β-Tubulin, the binding to stable MTs and the ability in increasing MT assembly lead us to propose Centaurin-α_2_ as a new MAP. We speculated that the strong interaction between Centaurin-α_2_ and MTs might also be involved in the translocation of Centaurin-α_2_ from cytoplasm to plasma membrane by PIP_2_ binding, after EGF stimulus [3]. Similarly phospholipase C-γ_1_ (PL C-γ_1_), which also contains a PH domain, was found to be constitutively associated with β-Tubulin and after EGF stimulation leads both proteins to move to membrane [20]. The results provided in both this study and in the previous work on PL C-γ_1_, are consistent with the reported involvement of PH domains of signalling molecules in their targeted translocation to cell membrane [21].

Centaurin-α_2_ overexpression results in a higher amount of acetylated Tubulin that correlates with MT stability probably contributing to modulate intracellular trafficking. Indeed, MT acetylation speeds up synaptic vesicles transport and the inhibition of HDAC6, a MT deacetylases [22], leads to Kif5-cargo delocalization [23]. Furthermore, it is reported that Centaurin-α_1_, that displays high homology with Centaurin-α_2_, interacts with Kif13B and it is then transported to the leading edge of the cell. [24]. Taken together, these findings suggest a possible mechanistic explanation for the Centaurin-α_2_ translocation, namely the modulation of Tubulin/MT posttranslational modifications (PTMs). On the other hand, no MT PTM has been more frequently equated with MT stability than acetylation, especially the acetylation of lys 40 on α-Tubulin, so much that the term “stable MTs” is often used almost synonymously with “Ac MTs” [8]. Nevertheless, Tubulin acetylation could be not the cause of MT stabilization [25] but it can occur on intrinsically more stable MTs, and both of them could be the cause or the consequence of intradimer conformational changes. Our results highlight that Centaurin-α_2_ interacts with cold- and nocodazole-resistant MTs and enhances acetylated-MTs in cell, but it directly stabilizes MTs in vitro. Therefore, we can propose that Centaurin-α_2_ directly contributes to the regulation of MT stability, particularly enhancing cold-adapted MT fraction, and to the exertion of the cell function that depends on acetylated Tubulin and stable MTs.

Although there is no a clear demonstration of the impact of acetylation of lys 40 of α-Tubulin on microtubular and cellular functions, it is known that MT stability correlated to MT acetylation, confers resistance to breakdown of the mitotic apparatus [10]. Given that Centaurin-α_2_ overexpression has been demonstrated to induce changes in MT stability, an effect on mitotic apparatus assembly was expected. The obtained results support this hypothesis as we observed a decreasing number of Hela cells showing aberrant mitotic spindles, when Centaurin-α_2_ is overexpressed, while the cell number increases when *ADAP2* gene is downregulated, even if the moderate endogenous expression of Centaurin-α_2_ in HeLa cells probably does not allow to appreciate a high deviation of the number of normal/abnormal spindle in the siRNA treated in relation to control cells. The primary function of centrosomes is to nucleate and anchor MTs, meaning they play a key role in the establishment of the interphasic MT network and bipolar mitotic spindles. Looking at centrosomal proteins involved in controlling centrosome function, several MAPs have been implicated in MT dynamics regulation [26], [27]. Among the others, Centrobindin is a centrosomal protein working as a MT stabilizing factor, able to promote MT formation, required for proper spindle formation during mitosis, and leading to defects in mitotic spindle when lacking [27]. Interestingly, our findings are consistent with such a peculiar role showing that Centaurin-α_2_ is a MAP, increases MT stability, and promotes the proper assembly of mitotic apparatus. Furthermore, the direct interaction of Centaurin-α_2_ with RanBPM [17], a known centrosomal protein [28], and the significant decreased number of centrosomes in Centaurin-α_2_ overexpressing cells, here reported, suggest its potential role in the control of centrosome formation. The integrity of centrosomes as well as their duplication are finely controlled by several factors including multiple kinases and oncogene activities [29], [30], [31]. In agreement with our results, a recent paper [32] suggests that modulation of MT stability is a regulatory element in the control of centrosome separation showing that the destabilization of interphase MTs speeds up this process. Thus, we propose that the Centaurin-α_2_-dependent stabilization of MTs contributes to the proper control of centrosome integrity and avoids centrosome amplification and the consequent formation of aberrant mitotic spindles. The presence of an aberrant number of centrosomes is reported in different tumor cells both *in vitro* and *in vivo* [33], [34] affecting mitotic spindle formation [29] and chromosome segregation for the presence of multinucleated cells [31] or a chromatin aggregation with one centrosome inside [11]. The occurrence of multipolar or the monopolar spindles as well as the aberrant number of centrosomes observed in HeLa and in other tumor cell lines, might be caused by a mutation and/or expression dysregulation of one or more genes encoding MAPs. The overexpression of Centaurin-α_2_ seems to show a protective effect on this aberrant condition by stabilizing MTs. Interestingly CENTA2 gene, alias *ADAP2*, was found to be expressed in neurofibromas and it has been candidate as a modifier gene contributing to a neurofibroma aberrant growth, both in number and in size, detected in NF1 microdeletion patients carrying a CENTA2 constitutional deletion [35]. Thus our results might address studies aimed at evaluating expression of Centaurin-α_2_ in tumors also suggesting to consider this protein as a new pharmacological molecule or target.

The expression of Centaurin-α_2_ during embryo development in central nervous system and in heart [4], might be related to the control of cytoskeleton stability during cell differentiation, shaping and migration. MT-based cytoskeleton is the primary spatial regulator of cell shape, and the coordinated action of Centaurin-α_2_ and its interactors might mediate changes in MT arrangement and MT-dependent function. Since RanBPM, which interacts with Centaurin-α_2_ is also found to be associated with Integrin at the plasma membrane [36], the coordinate function of Centaurin-α_2_ and RanBPM could promote the nucleation and polymerization of MTs at centrosomes, and could increase the stiffness of molecular scaffold at the point of cellular adhesion, mediating changes in cell shape. MT- and actin-based cytoskeleton act in concert to orchestrate cell remodelling; moreover, one MT function is to shape Actin cytoskeleton at specific location, i.e. cell cortex, through the transport or activation/inactivation of Actin regulators [37]. In the light of these results, we hypothesize that Centaurin-α_2_ behaves as cytoskeleton cross-talker since it interacts with MTs stabilizing them, as we showed here, and regulates actin reorganization via ARF6 [3]. Consequently, Tubulin and Centaurin-α_2_ interaction not only increases MT stability but it can also serve to modulate Actin reorganization, regulating the global cellular morphology. Furthermore, fine regulation of cytoskeleton could be relevant from a pathogenic point of view. We thus speculate that haploinsufficiency or loss of function mutations of *ADAP2* might affect MT stability and function of those cells, such as myocytes and neurons, in which the impairment of cytoskeleton organization, may lead to the onset of cardiovascular malformation and/or cognitive defects in NF1 microdeletion syndrome or in specific congenital diseases [5].

Evidence here provided on the binding between Centaurin-α_2_ and Tubulin-β C-ter region, which has been demonstrated to interact with MAPs [13], [14], and the increased MT stability detected after Centaurin-α_2_ overexpression, mimicking a typical MAPs' function [38], strongly indicates that Centaurin-α_2_ may be a novel MAP. Further studies aimed at investigating its function in tumoral cells and its role during development, may elucidate its physiological role together with its implication in both tumorigenesis and in the onset of some congenital diseases.

## Materials and Methods

### Yeast two-hybrid assay

Yeast two-hybrid assay has been carried out on *S. cerevisiae* L40, grown in yeast peptone dextrose medium (20 g/l tryptone, 10 g/l yeast extract) with dextrose (Clontech, Mountain View, CA) added with adenine hemisulfate (100 mg/l) (Sigma-Aldrich, St. Louis, MO). The bait, expressing Centaurin-α_2_, has been generated following PCR amplification of *ADAP2* cDNA – Image 5214358 gene – service –, with primers designed to introduce proper restriction site for BamHI, and then subcloned in pSTT91 vector, in frame with DNA binding domain sequence. To verify the occurrence of bait auto-activation, that should be excluded, L40 yeast has been co-transformed by lithium acetate in presence of polyethylene glycol solution (50% polyethylene glycol 3350 (Sigma-Aldrich), 10X Tris-EDTA, 10X lithium acetate) with the bait construct pSTT91-Centaurin-α_2_ and the pACT2 empty vector. Colonies grown on selective medium -Leu –Trp have been tested for the β-galactosidase assay, as well as the positive control L40 yeast transformed with two interacting proteins CoRest-Kia0601 (Figure S3). L40 has been transformed using 240 μg of pSST91-Centaurin-α_2_), 180 μg of human fetal brain cDNA library (Matchmaker, Clontech) (made by 3.5×10^6^ independent clones and constituted by cDNAs cloned in pACT2 vector using EcoRI and XhoI restriction enzimes, producing a fusion protein with regulatory protein GAL4), 600 μg of salmon sperm. The transformation has been plated on -Leu –Trp –His plates (yeast nitrogen base (6.7 g/l) (Sigma-Aldrich), 50% glucose, -Leu –Trp –His (0.62 g/l) (Clontech Laboratories Inc.) and agar (15 g/l) for 2–3 days at 30°C. Grown colonies have been screened for β-galactosidase activity using 67% X-Gal (20 mg/ml)/Z buffer pH 7 (16.1 g/l Na_2_HPO_4_7H_2_0; 5.5 g/l NaH_2_PO_4_H_2_O; 0.75 g/l KCl; 0.246 g/l MgSO_4_7H_2_O)/0.27% β-mercaptoethanol solution and nitrocellulose filters PROTRAN (Schleicher&Schuell BA 85) by incubation at 30°C, in presence of a positive control pBTM116-CoRest and pACT2-Kiaa0601. From colonies positive to β-galactosidase assay, prey plasmids have been extracted using lysis buffer (2% Triton-X-100, 1% SDS, 100 mM NaCl, 10 mM Tris pH 7.5, 1 mM EDTA), phenol:chloroform:isoamilic 25∶24∶1 and acid washed glass beads (G-8772, 425–600 μm, Sigma-Aldrich) and used for electroporation of *E. coli* Top 10. The plasmid, extracted using the Pure Yield Plasmid Miniprep System (Promega, WI), has been sequenced using the Big Dye^TM^ Terminator Cycle Sequence Ready Reaction Kit (Applied Biosystem, Carlsbad, CA), under the manufacturer's conditions, and resolved on a 3100 ABI Prism Genetic Analyzer (Applied Biosystem). The obtained cDNA, identified by http://blast.ncbi.nlm.nih.gov/Blast.cgi, has been digested with EcoRI (New England Biolabs, Ipswich, MA) and XhoI (New England Biolabs) restriction enzymes for identification of its length. Identified preys have been cotransformed in L40 yeast with different baits not interacting with them, pBTM116-CoRest, pBTM116-Laminin and pBTM116-Bars, in which every cDNA is cloned in frame with DNA binding domain, plated on -Leucine – Tryptophan plates (0.64 g/l) (Clontech Laboratories Inc) and incubated for 2-3 days at 30°C; the specificity of interaction with Centaurin-α_2_ has been observed after β-galactosidase assay.

### Coimmunoprecipitation assay

HeLa cells have been transfected with pCGN-Centaurin-α_2_ vector. After 48 hours cells have been washed with 10 ml of chilly 1X PBS in ice, scraped and centrifuged 5 minutes at 4°C at 180 g; the pellet has been resuspended in 5 volumes of lysis buffer (50 mM Tris pH 8, 150 mM NaCl, 0.5 mM EDTA, 10 mM imidazole pH 7, 0.5% Triton-X-100, 10% glycerol, 0.5 mM dithiothreitol, threo-2,3-dihydroxy-1,4-dithiolbutane, 1X protein inhibitor cocktail (Sigma-Aldrich), 1X phenylmethanesulfonyl fluoride), rotated 30 minutes at 4°C and centrifuged at 14000 g for 30 minutes at 4°C. The supernatant protein amount has been evaluated by Bradford Biorad protein assay (Biorad, Hercules, CA). The preparation of CoIP sample has been carried out as follow: 2.5 mg of total protein extracts have been added to 1.25 μg of HA-probe (Y-11) rabbit polyclonal IgG (Santa Cruz Biotechnology, Santa Cruz, CA) in a final volume of 300 μl of lysis buffer. The preimmune sample was made combining 0.5 mg of total protein extracts, 1.25 μg of rabbit IgG (Santa Cruz Biotechnology) in a final volume of 300 μl of lysis buffer.

Forty μl of slurry beads Protein G agarose (Invitrogen) in curran's blocking buffer buffer (10 mM MgCl, 100 mM KCl, 1 mM CaCl, 10 mM imidazole pH 7, 5% BSA, 0.3% Tween-20, 0.02% Na azide) have been added to the samples rotated at 4°C over night After a centrifugation of 2 minutes at 280 g at 4°C to pull down beads, the pellet has been washed with 1 ml of wash buffer (50 mM Tris pH 8, 150 mM NaCl, 10 mM imidazole pH 7, 0,1% Triton-X-100, 5% glycerol, 0.5 mM dithiothreitol, threo-2,3-dihydroxy-1,4-dithiolbutane, 1X protein inhibitor cocktail (Sigma-Aldrich), 1X phenylmethanesulfonyl fluoride) by rotating samples slowly for 4 minutes and following a centrifugation of 1 minutes at 280 g. Samples have been analyzed by 8% SDS-PAGE.

### Western blotting

The fractionation of cellular proteins into a soluble pool and a cytoskeleton associated pool was performed on HeLa cells, eventually transfected with pCGN-Centaurin-α_2_ or pCGN vectors, according to [39]. Briefly, cells were rinsed twice in 85 mM Pipes (pH 6.94), 10 mM EGTA, 1 mM MgCl_2_, 2 M glycerol, 1 mM phenylmethanesulfonylfluoride, 0.1 mM leupeptin, 1 μM pepstatin, 2 μg/ml aprotinin buffer, extracted for 10 min at room temperature with the same buffer containing 0.1% Triton-X-100. The extraction of nocodazole-treated cells was performed in buffer supplemented with nocodazole at the same concentration as the treatment to prevent repolymerisation of MTs during extraction. Similarly, cold-treated cells were extracted with buffer maintained at 4°C. After extraction, the Triton-X-100-soluble fractions were diluted 3∶1 with 4X SDS-PAGE sample buffer. The insoluble material remaining attached to the dish was scraped into SDS-PAGE sample buffer containing protease inhibitors.

For preparation of whole-cell extracts, HeLa cells, eventually transfected with pCGN-Centaurin-α_2_ or pCGN vectors, have been washed twice with 1X PBS and scraped into SDS-PAGE sample buffer containing protease inhibitors. The protein concentration has been determined by bicinchoninic acid reagent assay (Micro BCA, Pierce) and compared to a bovine serum albumin standard curve, to separate an equal amount of each sample.

Protein samples were separated by 7.5% SDS-PAGE, Western blotted onto nitrocellulose membrane (Whatman protran BA 85, 0.45 µm) (for coimmunoprecipitation experiments) or onto polyvinylidene fluoride membranes (ImmobilonTM-P, Millipore). Nitrocellulose membranes have been blocked with 5% BSA/1% 100X Na Azide in Tris-buffered saline for 30 minutes at room temperature; polyvinylidene fluoride membranes blocking has been performed with 0.1 M Tris-HCl/0.9% NaCl/5% BSA/0.05% Tween-20 for 150 minutes at room temperature. The membranes have been probed over night at 4°C with the following antibodies (Ab): diluted 1∶200 HA-probe (Y-11) rabbit polyclonal IgG (Santa Cruz Biotechnology) or diluted 1∶5000 monoclonal anti-β-Tubulin mouse IgG1 (clone TUB 2.1, Sigma-Aldrich) in Tris-buffered saline/5% BSA/0.1% Tween-20; diluted 1∶1000 α-Tubulin mouse IgG (clone B-5-1-2, Sigma-Aldrich), diluted 1∶1000 Ac-Tubulin mouse IgG (clone 6-11B-1, Sigma-Aldrich) or diluted 1∶1000 Tyr-Tubulin mouse IgG (clone TUB-1A2, Sigma-Aldrich) in 1X Tris-buffered saline/1% BSA/0.05% Tween-20. The incubation with secondary antibodies for 1 h at room temperature have been performed using the following antibodies: diluted 1∶2000 ECL-rabbit IgG, HRP (horseradish peroxidise)-linked whole Ab from donkey (Amersham, Piscataway, NJ) or 1∶1000 or goat anti-mouse IgG-HRP (Santa Cruz Biotechnology) in Tris-buffered saline/5% BSA/0.1% Tween-20 for 1 h at room temperature; diluted 1∶40000 HRP goat anti-rabbit IgG (Pierce, Rockford, IL) or diluted 1∶20000 HRP goat anti-mouse IgG (Sigma-Aldrich) in 1X Tris-buffered saline/1% BSA/0.05% Tween-20. Chemiluminescent signals have been detected using kit Supersignal West Pico Chemiluminescent Substract (Pierce), as instructed by the supplier. Protein quantification has been performed by scanning immunoblots with the JX-330 color image scanner (Sharp Electronics Europe) and analyzed by ImageJ software (National Institute of Health).

### Immunofluorescence and colocalization analyses

After 48 h HeLa cells, transfected with pCGN-Centaurin-α_2_ or pCGN vectors, were fixed with 4% paraformaldehyde (Sigma-Aldrich) for 10 minutes at room temperature and permeabilized for 4 min with 0.1% Triton-X-100/1X PBS. Some slides were extracted before the fixation to remove unassembled Tubulin. Briefly, cells were washed twice with PEM (Pipes 80 mM, EGTA 5 mM, MgCl_2_ 1 mM, pH 6,8, with protease inhibitors) and extracted 2 minutes with PEM buffer with Triton X-100 0,5%, NaCl 0,2 M protease inhibitors and 10 µM Paclitaxel (Sigma-Aldrich), and then fixed with methanol at −20°C. The samples have been treated with 5% BSA for 15 minutes at room temperature and incubated with diluted 1∶200 HA-probe (Y-11) rabbit polyclonal IgG (Santa Cruz Biotechnology), with diluted 1∶500 α-Tubulin mouse IgG (clone B-5-1-2, Sigma-Aldrich) or with diluted 1∶1000 γ-Tubulin mouse IgG (clone GTU-88, Sigma Aldrich) in Tris-buffered saline/1% BSA for 1 h at 37°C. After washing twice with 1X PBS, samples have been incubated with diluted 1∶1000 Alexa Fluor^TM^ 488 goat anti-rabbit (Invitrogen) and diluted 1∶1000 Alexa Fluor^TM^ 594 goat anti-mouse (Invitrogen) in Tris-buffered saline/1% BSA for 45 minutes at 37°C. Concurrent nuclear staining was made with 4′,6-Diamidino-2-phenylindole dihydrochloride (Sigma-Aldrich). The coverslips were mounted in Mowiol® (Calbiochem)– DABCO (Sigma-Aldrich) and examined with an Axiovert 200 M microscope (Carl Zeiss, Oberkochen, Germany) or with a confocal laser scan microscope imaging system (TCS SP2 AOBS, Leica Microsystems) equipped with an Ar-Ar/Kr488 nm, 561 nm and 405 nm diode lasers. Photomultiplier gain for each channel was adjusted to minimize background noise and saturated pixels and, once defined for control conditions, parameters were kept constant for all acquisitions; complete ‘z’ series optical sections were collected and projected onto a single plane using Leica TCS software. The analyses of colocalization parameters, reviewed in Bolte and Cordeliers (2006), have been made on the single-plane the raw images using the JACoP plug in for the ImageJ software.

Two different kinds of analyses were carried out: estimation of Pearson's coefficient, that represents the rough association between the two signals, and hence between two proteins, being the simpler way of measuring the dependence of pixels in dual-channel images, but particularly subjected to noise effect; evaluation of Manders' coefficients, that are good indicators of the relative distribution of the signals/proteins, representing the proportion of a signal (i.e. green) coincident with the other signal (i.e. red) over its total intensity, which may be applied even if the intensities in the two signals are really different.

### Recombinant centaurin-α2 production

In order to generate a plasmidic construct for the synthesis of human centaurin-α2 in *Escherichia coli*, the insert of the pCGN-based construct was amplified by PCR using the two primers 5′-GGTGGTCATATGGGCGATCGCG-3′ and 5′-GGTGGTGGATCCCTTCTTCCCACAAGGAG-3′, which include the *Nde*I and *Bam*HI restriction sites (underlined), respectively. After the restriction enzyme digestion, the resulting fragment was inserted between the same sites of the pET28b vector (Novagen), yielding the expression plasmid pET-Centα2, whose insert region was fully sequenced to exclude the presence of PCR artifacts. For recombinant centaurin-α2 production, BL21(DE3) *E. coli* cells (Novagen) were cotransformed with pLacIRARE2 (Novagen) and pET-Centα2, grown in liquid terrific broth supplemented with chloramphenicol and kanamycin, and induced overnight at 15°C with 0.1 mM isopropyl-β-D-thiogalactopyranoside. The target protein, which carries an N-terminal His-tag extension, was isolated from the soluble fraction of the crude bacterial lysate by immobilized metal ion affinity chromatography on a His Trap HP 5 ml prepacked column (GE Helthcare), according to the manufacturer directions. After concentration by ultrafiltration and desalting by gel filtration, the protein, showing higher than 95% purity by SDS-PAGE, was subdivided in aliquots and stored at −70°C until needed.

### Tubulin purification and assembly assay

Tubulin was purified from bovine brain purchased from a local slaughterhouse, conserved before use in ice-cold PIPES buffer (1 M K-PIPES, pH 6.9, 2 mM EGTA, and 1 mM MgCl_2_) and used as soon as possible. Pure tubulin was obtained by two cycles of polymerisation-depolymerisation in a high-molarity buffer [40], and protein concentration was determined by the MicroBCA assay kit (Pierce). To remove the cold-stable fraction, just before the use, tubulin was centrifuged at 100000 g (Beckmann, rotor TLA 100.3) for 30 minutes at 4°C. Assembly buffer (80 mM K-PIPES pH 6.9, 2 mM EGTA, 1 mM MgCl_2_, and 10% glycerol) contained tubulin at two different concentrations, 40 µM (standard conditions) or 18 µM (around the critical concentration) and 5 µM Centaurin-α_2_, and the polymerization run for 90 minutes at 37°C. As control, tubulin was incubated with the same buffer containing Centaurin-α_2_. In some cases, after the assembly samples were kept on ice for 30 minutes, to allow microtubule depolymerisation. To separate microtubules (pellet) and tubulin dimers (supernatant), samples were centrifuged at 16500 g for 30 minutes at room temperature; equal amount of each fraction was resolved on 10% SDS-PAGE gel and stained with Comassie Blue. Alternatively, at the end of the assembly, microtubules were fixed with 0.5% glutaraldehyde in BRB 80 and put onto poli-L-lysine coated slides. Subsequently, the presence of His-tagged Centaurin-α_2_ was detected by using an anti-His primary antibody (IgG mouse, clone 4A12E4, Invitrogen) and Alexa Fluor™ 488 goat anti-mouse (Invitrogen). The coverslips were mounted in Mowiol® (Calbiochem, San Diego, CA)–DABCO (Sigma-Aldrich) and image acquisition was performed using a Zeiss Axiovert 200 equipped with differential interference contrast (DIC) optics and fluorescent filter units, a 63× oil objective, and a digital image recording system (Axiocam HRM Rev. 2 camera driven by Axiovision software rel. 4.4, Zeiss).

### Mammalian cell cultures and transfection

HeLa cells have been cultured in Dulbecco's modified Eagle's medium with 10% fetal bovine serum, 100 U/ml penicillin-streptomycin and 0.01 mM L-glutamine (EuroClone, Pero MI). Cultures have been maintained at 37°C in a 5% CO_2_ incubator. Cells have been transfected at 60–70% confluence and cultured at 37°C in a humidified atmosphere with 5% CO_2_. Transfection has been performed with LIPOFECTAMINE 2000 (Invitrogen) according to the manufacturer's instructions using pCGN vector or pCGN-Centaurin-α_2_ (generated from PCR amplification of *ADAP2* cDNA – Image 5214358 gene – service – with primers designed to introduce proper restriction site for BamHI and then by subcloning them in pCGN vector, in frame with HA sequence).

In experiments using nocodazole (3 μg/ml) (Sigma-Aldrich) to depolymerize MTs, the drug was diluted into the culture medium and incubated for 3 h at 37°C prior to cellular fractionation for biochemical analyses. Cold treatments were performed at 4°C by incubating cultures in a refrigerator for 3 h prior to cellular fractionation for biochemical analyses.

### *ADAP2* silencing

*ADAP2* gene silencing has been carried out using 50 nM ON-TARGET plus SMART pool siRNA Centa2 (J-020444-09: gaguuaaaucugugcgacu; J-020444-10: aaguaugagagacgggaau; J-020444-11: cuucaagcaccacgcagaa; J-020444-12: gauaugaagccuacgaaga) and ON-TARGET plus Non-targeting Pool (100 nM) (Thermo SCIENTIFIC, CO, USA) with LIPOFECTAMINE 2000 (Invitrogen, Carlsbad, CA), as described [41]. The total RNAs from both treated and non-treated cells were isolated at 24, 48 and 72 hours after the transfection using TRIzol reagent (Invitrogen), according to the manufacturer's instructions. Purity of RNAs (A260/A280 value of 1.8–2.1) and concentration were measured using Nanodrop spectrophotometer. To eliminate DNA contamination, total RNAs were treated with DNase I (Invitrogen). cDNA was synthesized from total RNA (1 µg) in 20 µl reactions, using iScript cDNA Synthesis Kit from BioRad (Hercules, CA, USA).

To estimate the mRNA amount a three-step real-time PCR analysis was performed, by using Gotaq qPCR sybergreen (Promega, WI), Centa 2 and HPRTI housekeeping primer forward and reverse (Centa2 fw acaccaggaacctgtttgtgt; Centa2 rev: gagggcattgaaccagtcc; and HPRTI fw: tgacactggcaaaacaatgca, HPRTI rev: ggtccttttcaccagcaagct).

### Statistical analysis

The statistical significance of treatment has been assessed by Student's t-Test for parametric variables and by χ^2^ test for non-parametric ones, and analyses were performed using STATISTICA (StatSoft Inc., Tulsa, OK). All experiments have been repeated at least three times and data have been expressed as means ± S.E.M (Standard Error of the Mean).

## Supporting Information

Figure S1 Centaurin-α2 mRNA expression level in HeLa and H937 cell. Centaurin-α2 mRNA expression level in HeLa and H937 cell lines by real time RT-PCR normalized with the GAPDH transcript housekeeping. The level of GAPDH expression was comparable in all samples tested. Following the mean value of Centaurin-α2 expression level, calculated by GAPDH normalization and the estimation of 2^−ΔCt^, is about 20 fold lower in HeLa than in H937 cell lines.
(TIF)

Figure S2 Centaurin-α_2_ colocalizes with MTs. Fluorograms showing the degree of colocalization between green (HA) and red (MTs) signals in pCGN (pCGN) or pCGN-Centaurin-α_2_ (pCenta) expression.
(TIF)

Figure S3 Bait autoactivation assay. β-galactosidase assay of L40 yeast co-transformed with the bait construct pSTT91-centaurin-α_2_ and the pACT2 empty vector, and of the positive control L40 yeast transformed with the two known interacting proteins CoRest-Kia0601. The presence of blue colonies only in the control transformed L40-yeast indicates that the bait pSTT91-centaurin-α_2_ does not auto-activate the β-galactosidase gene.
(TIF)
