# Supplementary figures and images for: Centaurin-α2 Interacts with β-Tubulin and Stabilizes Microtubules
Source: PLoS One. 2012 Dec 20;7(12):e52867. doi: 10.1371/journal.pone.0052867 (PMC3527619; doi:10.1371/journal.pone.0052867)

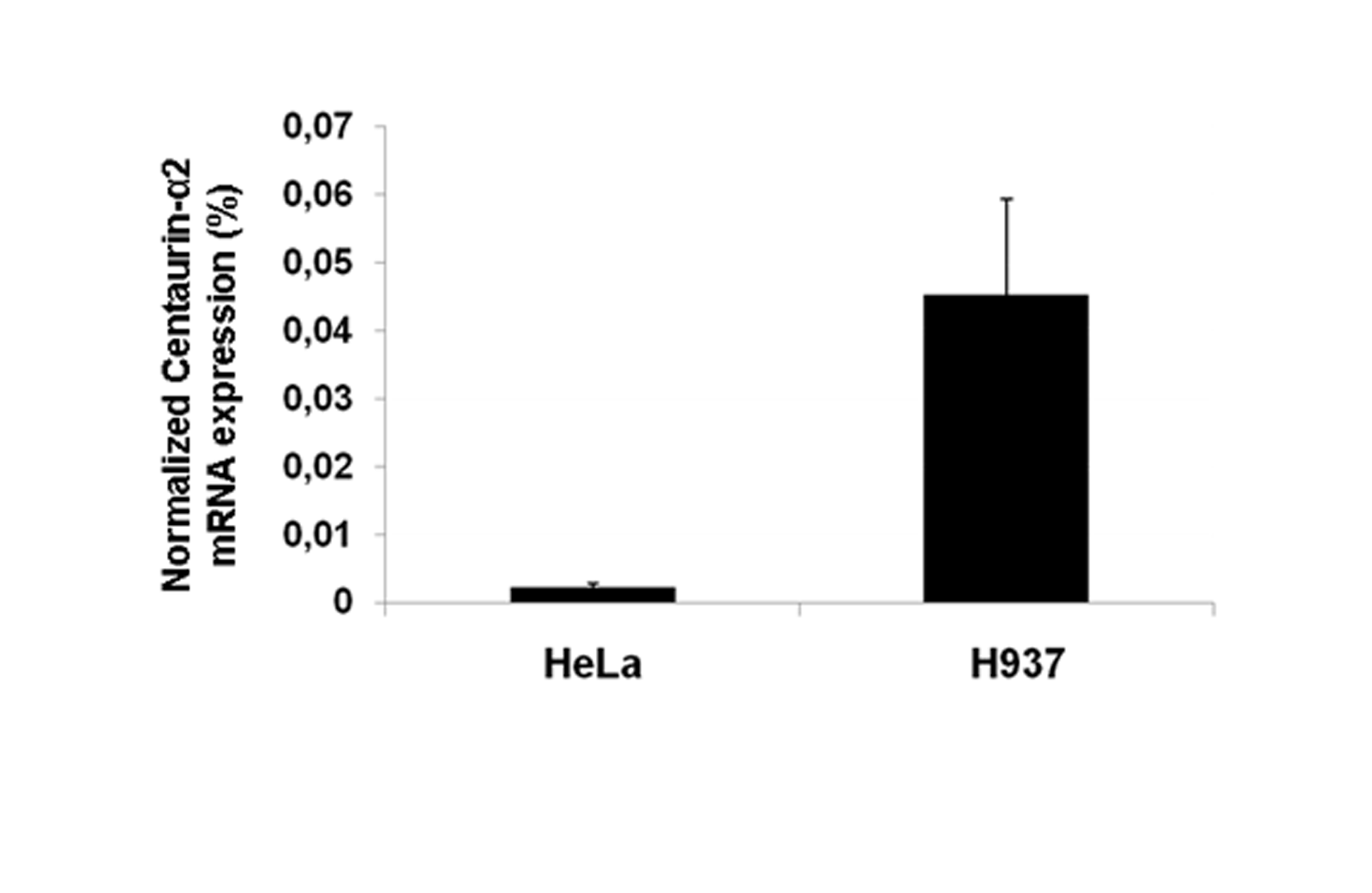

Supplement: Figure S1 — Centaurin-α2 mRNA expression level in HeLa and H937 cell. Centaurin-α2 mRNA expression level in HeLa and H937 cell lines by real time RT-PCR normalized with the GAPDH transcript housekeeping. The level of GAPDH expression was comparable in all samples tested. Following the mean value of Centaurin-α2 expression level, calculated by GAPDH normalization and the estimation of 2−ΔCt, is about 20 fold lower in HeLa than in H937 cell lines. (TIF) [file pone.0052867.s001.tif]

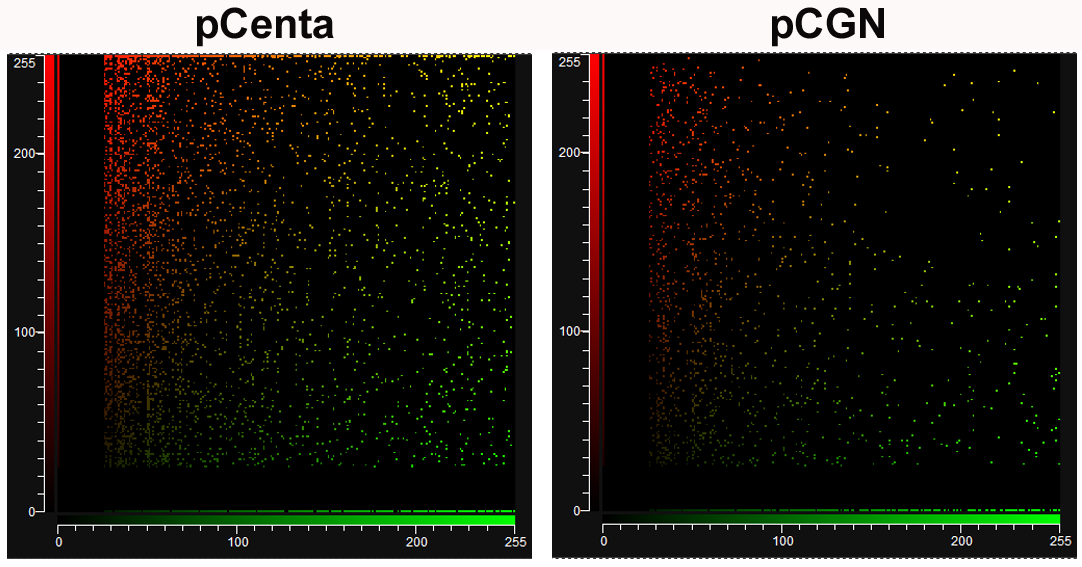

Supplement: Figure S2 — Centaurin-α2 colocalizes with MTs. Fluorograms showing the degree of colocalization between green (HA) and red (MTs) signals in pCGN (pCGN) or pCGN-Centaurin-α2 (pCenta) expression. (TIF) [file pone.0052867.s002.tif]

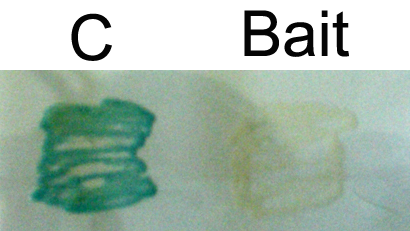

Supplement: Figure S3 — Bait autoactivation assay. β-galactosidase assay of L40 yeast co-transformed with the bait construct pSTT91-centaurin-α2 and the pACT2 empty vector, and of the positive control L40 yeast transformed with the two known interacting proteins CoRest-Kia0601. The presence of blue colonies only in the control transformed L40-yeast indicates that the bait pSTT91-centaurin-α2 does not auto-activate the β-galactosidase gene. (TIF) [file pone.0052867.s003.tif]
